# Colinearity and Similar Expression Pattern of Rice *DREB1s* Reveal Their Functional Conservation in the Cold-Responsive Pathway

**DOI:** 10.1371/journal.pone.0047275

**Published:** 2012-10-16

**Authors:** Donghai Mao, Caiyan Chen

**Affiliations:** Key Laboratory of Agro-Ecological Processes in Subtropical Region, Institute of Subtropical Agriculture, Chinese Academy of Sciences, Changsha, China; National Taiwan University, Taiwan

## Abstract

The clustered genes C-repeat (CRT) binding factor (*CBF)1/* dehydration-responsive element binding protein *(DREB)1B*, *CBF2/DREB1C*, and *CBF3/DREB1A* play a central role in cold acclimation and facilitate plant resistance to freezing in *Arabidopsis thaliana*. Rice (*Oryza sativa* L.) is very sensitive to low temperatures; enhancing the cold stress tolerance of rice is a key challenge to increasing its yield. In this study, we demonstrate chilling acclimation, a phenomenon similar to *Arabidopsis* cold acclimation, in rice. To determine whether rice *CBF/DREB1* genes participate in this cold-responsive pathway, all putative homologs of *Arabidopsis DREB1* genes were filtered from the complete rice genome through a BLASTP search, followed by phylogenetic, colinearity and expression analysis. We thereby identified 10 rice genes as putative *DREB1* homologs: nine of these were located in rice genomic regions with some colinearity to the *Arabidopsis CBF1*–*CBF4* region. Expression profiling revealed that six of these genes (Os01g73770, Os02g45450, Os04g48350, Os06g03670, Os09g35010, and Os09g35030) were similarly expressed in response to chilling acclimation and cold stress and were co-expressed with genes involved in cold signalling, suggesting that these *DREB1* homologs may be involved in the cold response in rice. The results presented here serve as a prelude towards understanding the function of rice homologs of *DREB1* genes in cold-sensitive crops.

## Introduction

Low temperature is one of the adverse environmental factors that can limit crop distribution and productivity. This adverse environment is particularly widespread in temperate regions, where a significant temperature change frequently occurs during the transition between cold and warm seasons. To overcome this constraint, most temperate plants have evolved an adaptive response, generally known as cold acclimation. Cold acclimation is a process by which plants acquire freezing tolerance upon prior exposure to low, non-freezing temperatures [1]. The molecular mechanism of cold acclimation may involve multiple pathways; of these, the CBF/DREB1-dependent cold-responsive pathway has been clearly elucidated and plays an important role in cold acclimation [2].

To date, the molecular mechanism of the C-repeat (CRT) binding factor*/* dehydration-responsive element binding protein 1 (CBF/DREB1)-dependent cold-responsive signal pathway has been studied in depth in the freezing-tolerant plant *Arabidopsis thaliana* [3]. Three *CBF* genes, *CBF1/DREB1B*, *CBF2/DREB1C,* and *CBF3/DREB1A*, play a central role in this pathway. These genes belong to a small subfamily of the ethylene response factor/Apetala2 (ERF/AP2) family, named the DREB1 family that contains six members (i.e. *CBF1*–*CBF4*, *DDF1*, and *DDF2*). Besides the ERF/AP2 domain, the six genes have special conserved domains or motifs (the nuclear localization signal [NLS], DSAWR, and LWSY motifs) [4]. These genes encode DREB1 proteins - transcription factors that recognise the C-repeat elements of cold-responsive genes (*COR*s) and activate their expression. *DREB1* genes are rapidly induced to high levels by low temperature treatment, subsequently activate *COR*s, and promote plant resistance to cold stress. All of the *CORs* with different functions are regulated by CBF/DREB1s; therefore, they are collectively called the ‘CBF regulon’ [5]. Three genes, *CBF1*–*CBF3,* are indispensable for the signal transduction of upstream cold-involved transcription factors. *Inducer of CBF expression 1* (*ICE1*) encodes a Myc-type basic helix-loop-helix (bHLH) transcription factor, which binds specifically to the Myc elements of *CBF3*, and induces its expression at low temperatures [6]. *ICE2*, a paralog of *ICE1*, positively regulates the expression of *CBF3* [7]. *MYB15* (an R2R3-MYB family protein) negatively regulates the expression of *CBF* genes by binding to their MYB elements [8]. A cold-induced C2H2 zinc finger transcription factor gene, *ZAT12*, also appears to function as a negative regulator of *CBF* gene expression. Transcriptome analysis of ZAT12-overexpressing *Arabidopsis* revealed that the ZAT12 regulon consists of at least 24 cold standard set (*COS*) genes [9]. In addition, calmodulin-binding transcription activator 3 (CAMTA3) binds to the CM2 elements of *CBF2* and positively regulates the expression of the latter [10]. In short, *Arabidopsis CBF1*–*CBF3* play central roles in the cold responsive pathway.

The functions of *CBF* homologs have been extensively studied in low temperature-sensitive plants, such as maize and rice, which are incapable of cold acclimation. However, theses two species do exhibit chilling acclimation, which is similar to the cold acclimation of *Arabidopsis*. Following exposure to suboptimal low temperatures for some time, the temperature threshold for chilling damage is lowered in freezing-sensitive plants such as maize [11] and rice (see our results). Thus, it has been proposed that *CBF* homologs are also required for chilling resistance in cold-sensitive plants. In rice, ten putative *CBF* homologs (*OsDREB1A* to *OsDREB1J*) have been identified. Of these, *OsDREB1A, OsDREB1B,* and *OsDREB1F* are induced, whereas *OsDREB1D* is not induced by cold stress. Over-expressions of *OsDREB1A*, *OsDREB1D,* and *OsDREB1F* can up-regulate the expression of *COR*s and enhance cold resistance in *Arabidopsis* [12, 13, 14, 15, and 16]. Tomato (*Solanum lycopersicon*) also has three clustered *CBF* homologs, *LeCBF1*–*LeCBF3*, but only *LeCBF1* was found to be cold-inducible. Constitutive overexpression of *LeCBF1* in transgenic *Arabidopsis* plants induced the expression of CBF-targeted genes and increased freezing tolerance. However, the constitutive overexpression of either *LeCBF1* or *AtCBF3* in transgenic tomato plants did not increase cold tolerance. Additionally, only four out of approximately 8000 tomato genes were induced 2.5-fold or more in plants overexpressing *LeCBF1* or *AtCBF3*; of these, three were putative members of the tomato CBF regulon, as they were also up-regulated in response to low temperature. Taken together, these results suggest that freezing-sensitive tomato has a functional CBF/DREB1-dependent cold-responsive pathway, but the tomato CBF regulon differs from that of freezing-tolerant *Arabidopsis* [17]. Thus, it can be concluded that the functions of *CBF* homologs are conserved, at least in part, in low temperature-sensitive plants.

Rice is one of the most important low temperature-sensitive grass crops with an available whole-genome sequence. To improve its cold tolerance, both forward and reverse genetics approaches have been adopted to identify the genes responsible for cold tolerance in rice. Several quantitative trait loci (QTLs) have been mapped or isolated using genetic populations in rice [18], [19], [20], [21], [22], and three *CBF* homologs have been identified to function in the cold-response pathway in the *Arabidopsis* genome background [12], [14], [15], [16]. However, a limited number of QTLs have been fine-mapped onto regions containing *CBF*/*DREB1* genes in the rice genome, and the functions of rice *CBF* homologs in the rice genome background remain to be elucidated. This raises the question of whether there are additional homologs of *Arabidopsis CBF* genes in the rice genome, and how these rice homologs function in response to cold treatment. To answer these questions, we searched the entire rice genome for homologs of *CBF/DREB1*s. A total of 10 rice genes were identified as putative homologs of *CBF* genes according to reciprocal blast search results, phylogenetic relationships, and protein sequence similarity. The results of our expression analysis and gene ontology (GO) enrichment analysis suggest that six of these genes (Os01g73770, Os02g45450, Os04g48350, Os06g03670, Os09g35010, and Os09g35030) may be involved in the cold response in rice, whereas other homologs may have acquired new functions or diverged and became pseudogenes. These results provide insights into the mechanisms of chilling tolerance in rice, as well as the evolution of *CBF* genes in monocots.

## Materials and Methods

### Phenotypes of Low-temperature Resistance in Rice Seedlings

The resistance of rice plants to low temperatures was determined by the survival rates of 7-day-old seedlings of four rice varieties (the indica varieties 9311 and Kasalath, and the japonica varieties Nipponbare and TP309) after cold stress with or without chilling acclimation. For cold treatment, 7-day-old seedlings were first kept for 4 days (in the case of indica 9311 and Kasalath varieties) or seven days (in the case of japonica Nipponbare and TP309 varieties) at a low temperature of 4°C, and then restored for 10 days to a normal growth temperature (28°C for the day/25°C for the night). For chilling acclimation treatment (CA), 7-day-old seedlings were initially kept for two days at a chilling temperature of 12°C, then for four days (indica rice, both 9311 and Kasalath) or seven days (japonica Nipponbare and TP309 varieties) at a low temperature of 4°C, and subsequently restored for 10 days to the normal growth temperature.

### Sequences of the Rice and *Arabidopsis* Genes

The sequences of *Arabidopsis* genes belonging to the *DREB1* subfamily were downloaded from the TAIR9 database (http://www.arabidopsis.org, TAIR9_pep_20090619). Conserved protein sequences of *DREB1s* (i.e. the NLS, the AP2/ERF domain, and the DSAW motif) were used as query sequences to carry out BLASTP searches against rice protein sequences in the Michigan State University (MSU) Rice Genome Annotation Project (RGAP) database, release 7 (http://rice.plantbiology.msu.edu/analyses_search_blast.shtml). The resulting sequences from BLASTP were downloaded from the Rice MSU Osa1 database, release 6.1 (http://rice.plantbiology.msu.edu). All protein sequences were subjected to phylogenetic analysis. Putative homologs were then further confirmed by conserved domain alignment and colinearity analyses.

### Identification of Rice Homologs of *Arabidopsis DREB1s*

In order to identify rice homologs of *Arabidopsis DREB1s*, the full protein sequences of all non-redundant candidates obtained from BLASTP and *Arabidopsis DREB1*s were used to construct a phylogenetic tree by the neighbour-joining method using the ClustalW2 software [23], [24]. From the phylogenetic tree, rice genes with close evolutionary relationships to *Arabidopsis DREB1*s were analysed by BLASTP against the *Arabidopsis* genome, and the genes possessing the highest E value or similarity with *Arabidopsis DREB1*s in the BLASTP analysis were ascertained to be candidate *DREB1* homologs. The full protein sequences of these genes were aligned using ClustalW2, and also used to construct a phylogenetic tree by the neighbour-joining method of ClustalW2. Bootstrap analysis was performed using 1000 replicates. The phylogenetic tree was displayed by MEGA 4.0 [25], and then edited and viewed by TreeView software [26].

### Colinearity Analysis between/among Regions Containing *Arabidopsis DREB1s* or their Rice Homologs

The colinearity between/among regions containing either *Arabidopsis DREB1s* or their rice homologs was analysed using reciprocal blast searches as previously described by Higgins et al [27]. Those genes belonging to small families, or existing as singleton genes, such as the gene adjacent to Os06g03670 (Os06g03600, encoding an SLK protein), were used in a BLASTP search against the protein database of *Arabidopsis*, and the genes with the highest E values and/or similarity scores in the results were taken as candidates for their orthologs. Further, protein sequences for these ortholog candidates were used in a BLASTP search of the rice protein database, and the rice genes with highest E values and/or similar scores in the result were then taken as the query genes in the first BLASTP. For some genes with multiple family members, such as genes belonging to the *ERF/AP2* family, orthologs between rice and *Arabidopsis* were determined based not only on the results of reciprocal blast searches but also on the conservation of neighbouring genes and order between the two species resulting from colinearity analysis [28].

### Microarray Expression Data Analysis

Expression profile data of rice *DREB1* genes for an indica variety, Zhenshan 97, were extracted from the Collection Rice Expression Profile (CREP) database (http://crep.ncpgr.cn) [29], which is composed of Affymetrix rice microarray data from the hybridization of RNA samples from 39 tissues/organs covering the entire life cycle of rice. Hierarchical cluster analysis was performed using the logarithms of the expression values of each gene and then a heat map was constructed using R software, version 2.9.0 (http://www.r-project.org).

Expression profile data for rice *DREB1s* under abiotic stresses such as salt, cold, and drought were extracted from the RiceGE database (http://signal.salk.edu/cgi-bin/RiceGE). The rice seedlings that were subjected to abiotic stresses were of the indica variety IR64 and were seven days old. According to the protocol, the rice seedlings were soaked in a 200 mM NaCl solution for 3 h for salt treatment, placed between folds of dry tissue paper at 28°C for 3 h for drought treatment, and kept at 4°C for 3 h for cold treatment.

### Expression Analysis of Rice *DREB1* Genes at Low Temperatures

Seven-day-old seedlings of the cold-sensitive indica variety Kasalath and the cold-resistant japonica variety Nipponbare were both subjected to low temperature treatments at 4°C and 12°C. Samples were collected at 0, 3, 6, 12, and 24 h timepoints during the treatments, and those from the 0 h timepoint were regarded as controls. Total RNA (2 µg) of each sample was isolated using an RNA extraction kit (TRIzol reagent, Invitrogen, Carlsbad, CA) and reverse-transcribed in a 20-µL reaction using Revert Aid™ First Strand cDNA Synthesis Kit (Fermentas, Ontario, Canada) according to the manufacturer’s instructions. Real-time polymerase chain reaction (PCR) was performed in a 10-µL reaction mixture with 1 µL of first-strand cDNA, 5 µL 2× SYBR® Premix Ex Taq™ (TaKaRa, Shiga, Japan), 0.2 µL 50× ROX reference dye, and 1 µL of each primer (2.5 µM). The reactions were carried out using an ABI PRISM 7500 system (Applied Biosystems, Foster City, CA). The following amplification protocol was used: 95°C for 30 s, followed by 40 cycles of 95°C for 5 s, and 60°C for 30 s. Rice *ubiquitin 5* was used as the internal control. The relative expression levels were analysed using the 2^−ΔΔCt^ method [30]. All experiments were repeated at least 3 times independently. The sequences of primers used in real-time PCR are shown in Table 1.

**Table 1:** The messages of relative genes in the study.

TIGR Locus	Gene	qPCR forward primers	qPCR reverse primers	Probe Set	EST/cDNA
Os01g73770	*OsDREB1F*	AGGACGCCATCTTCGACAT	GTCGAGAGATCTCCCAATCG	Os.40428.1.S1_at	J013070D13^a^
Os02g45450	*OsDREB1G*	CCCGTACTACGAGGTCATGG	GCTACCTACGGCAGGATCAC	Os.51078.1.S1_at	001–021-H10^a^
Os04g48350	*OsDREB1E*	GAATTCGAAATGCAGGGGTA	CTCGCAGTCGTAGTCCTCCT	Os.57527.1.S1_at	AY114110^b^
Os06g03670	*OsDREB1C*	CAAAGCTTATCAGCAGTAGC	GGTTAGTAGCAGAAAGACTTG	Os.4463.1.S1_s_at	AY327040^b^
Os06g06970	*OsDREB1D*	CAAAGCTTATCAGCAGTAGC	GGTTAGTAGCAGAAAGACTTG	Os.50638.1.S1_at	AY345235^b^
Os08g43200	*OsDREB1J*	CATGACCAGCTGCCCGACGT	GTGACAGAACGGGCGACGAC	OsAffx.6121.1.A1_at	CI268958^c^
Os08g43210	*OsDREB1I*	GAGCCTGTACTACGCGAGCTTA	TCAGCGATGTCGCTTGAGTC	OsAffx.29642.1.S1_at	CI261507^c^
Os09g35010	*OsDREB1B*	GATGGCGACGAAGAAGAAGA	GAACCTGAACCCGTCGTC	Os.5816.1.S1_at	001–102-G08^a^
Os09g35020	*OsDREB1H*			-	NO^d^
Os09g35030	*OsDREB1A*	ACCTGTACTACGCGAGCTTG	TAGTAGCTCCAGAGTGGGAC	Os.14125.1.S1_at	001–200-A04^a^
Os01g22490	*Ubiquitin 5*	ACCACTTCGACCGCCACTACT	ACGCCTAAGCCTGCTGGTT	-	CF293679^c^

a KOME FL-cDNA;

b Community cDNA;

c NCBI-EST;

d No EST or FL-cDNA found.

### GO Enrichment Analysis of Genes Co-expressed with Rice *DREB1* Homologs

Genes co-expressed with the rice *DREB1* homologs were determined by Pearson pair-wise correlation analysis, the results of which are already available in the CREP microarray expression database (http://crep.ncpgr.cn) and the Rice Oligo Array Database (ROAD; http://www.ricearray.org). Both CREP and ROAD databases were used to analyse gene expression profiles over the entire life cycle of rice growth and development. The genes co-expressed with the rice *DREB1*s were used in singular enrichment analysis (SEA) with the online software AgriGO (http://bioinfo.cau.edu.cn/agriGO/) [31]. Biological process was selected as the GO category, and the ‘Rice TIGR Gene model’ was selected as the background. Two types of co-expression databases, abiotic stress and general in ROAD, were taken for GO enrichment analysis using the Chi square test. All co-expressed genes for each rice *DREB1* gene were mapped to the GO category by the online GO enrichment analysis software, and Chi square tests for GO enrichment analysis were performed for genes that belong to ‘responsive to freezing (GO:0050826)’ as well as for genes that were mapped to both ‘responsive to freezing’ (GO:0050826) and ‘responsive to stress’ (GO:0006250), and were induced by a cold stress of 4°C for 3 h in the SALK RiceGE database.

## Results

### Rice Resistance to Cold Stress with or without Chilling Acclimation

**Figure 1:**
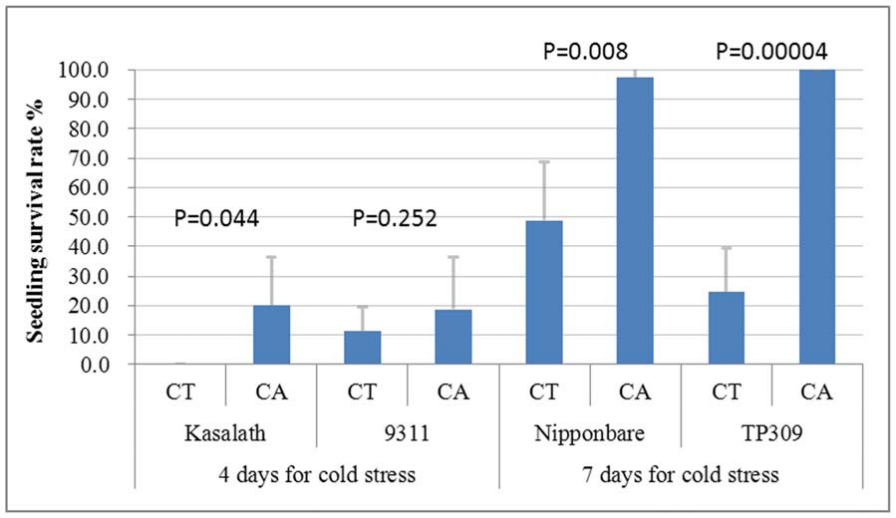
Survival rate of 7-day-old rice seedlings after the low temperature stress with or without chilling acclimation. Cold treatment (CT): 7-day-old seedlings were first maintained for 4 days (for indica rice: 9311 and Kasalath) or 7 days (for japonica rice: Nipponbare and TP309) at a low temperature of 4°C, then restored for 10 days to normal growth temperature. Chilling acclimation treatment (CA): 7-day-old seedlings were first maintained for 2 days at a mild temperature of 12°C, then 4 days (for indica rice: 9311 and Kasalath) or 7 days (for japonica rice: Nipponbare and TP309) at a low temperature of 4°C, and finally restored for 10 days to normal growth temperature. Each rice varieties were treated with four times of independent experiments and the bars represent the standard deviation. P values of T-test for difference between CT and CA treatments of each variety were shown in Fig. 1.

In order to determine whether rice exhibits chilling acclimation, 7-day-old seedlings of four rice varieties (indica, 9311 and Kasalath; japonica, Nipponbare and TP309) were treated with cold stress with or without chilling acclimation. Growth and survival in all varieties tested were improved by first exposing seedlings to a 12°C acclimation treatment for 2 d before applying the chilling treatment (Fig. 1). In the case of indica rice, approximately 20% of the acclimated seedlings of Kasalath, but none of the non-acclimated seedlings, were alive after cold treatment at 4°C for four days (t test, P<0.05). Enhanced cold resistance after chilling acclimation was also observed in indica rice 9311, though the difference was not statistically significant (P = 0.252). For japonica rice (i.e. Nipponbare and TP309), at least 97% of the acclimated seedlings, but only 24.5–49.6% of the non-acclimated seedlings were alive after cold treatment at 4°C for seven days (P<0.01). This suggests that rice possesses the ability to undergo chilling acclimation as described in maize [11].

Moreover, these results also showed that both the japonica rice varieties (i.e. Nipponbare and TP309) could survive more than two days of cold stress with more surviving seedlings than was seen in the two indica varieties (i.e. Kasalath and 9311), with or without chilling acclimation. For example, in contrast to only ∼20% of the acclimated indica rice seedlings surviving, 97–100% of the acclimated japonica rice seedlings survived. Even without chilling acclimation, 24.5–49.6% of japonica rice seedlings survived after a longer period of cold stress (4°C for seven days), but only 0–11% of indica rice seedlings were alive after a shorter period of cold stress (4°C for four days). These data indicate that japonica rice is more resistant to low temperature than is indica rice.

### Identification of Rice *DREB1* Homologs

**Figure 2:**
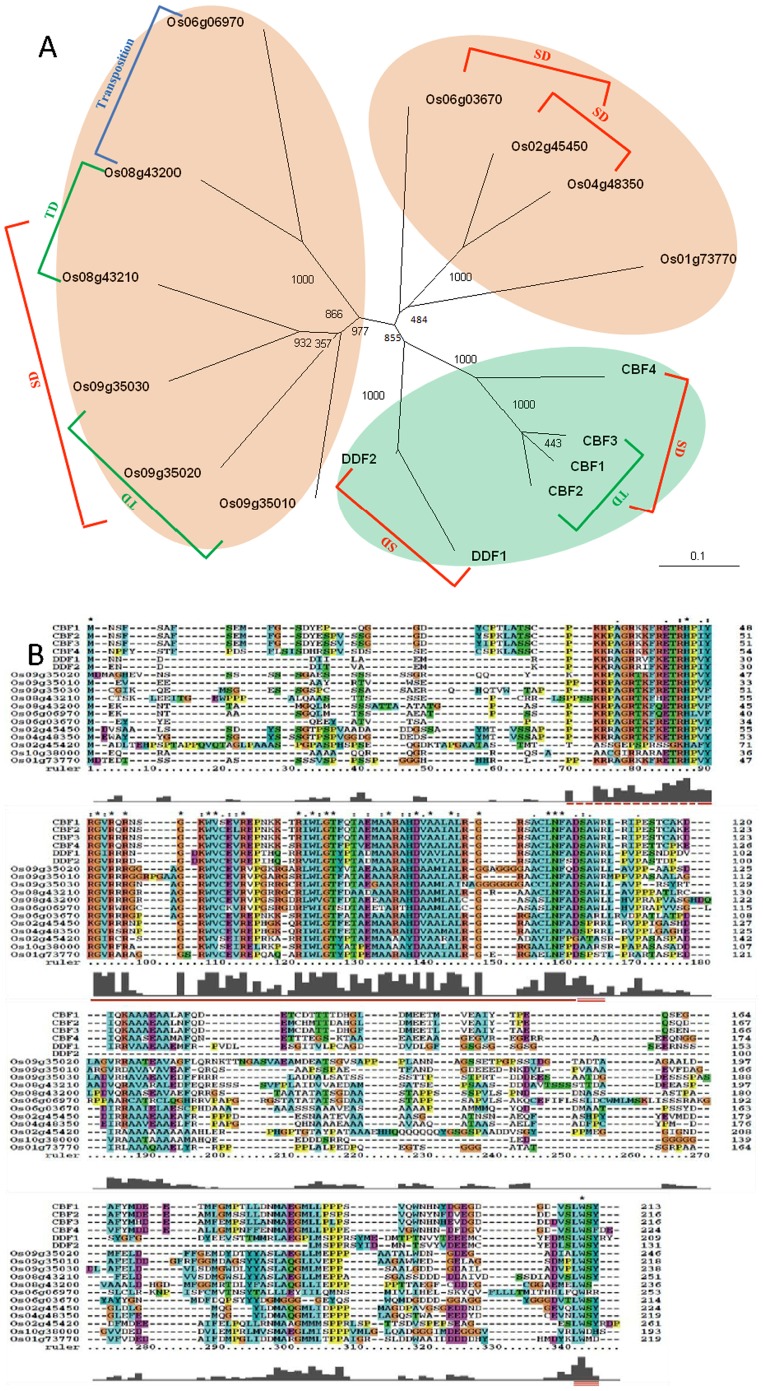
Phylogenetic analysis and sequence alignment of *Arabidopsis DREB1*s and their homologs in rice using ClustalW2. A: The phylogenetic analysis was carried out by the neighbour-joining method of ClustalW2, and the tree was edited and viewed by TreeView software. SD: segmental duplication; TD: tandem duplication; Transposition: single gene duplication by transposition. Bootstrap values from 1000 replicates were indicated at each node. Scale bar represented 0.1 amino acid substitution per site. B: The NLS (nuclear localization signal), ERF/AP2 domain, DSAW motif, and LWSY motif are shown. The alignment of the 141–171 region of *CBF1* and the corresponding regions of its rice and *Arabidopsis* homologs has been omitted due to the absence of conserved motif.

In the *Arabidopsis* genome, there are six genes belonging to the *CBF/DREB1* family, which is a small subfamily of the ERF/AP2 gene family. The *CBF/DREB1* family contains the *CBF1*–*CBF4, DDF1,* and *DDF2* genes, and each of the corresponding proteins has conserved sequences such as the nuclear localization signal (NLS), ERF/AP2 domain, and the DSAW and LWSY motives (Fig. 2). As the first three motifs or domains are arranged together, these sequences are suited to searching for homologous genes in rice using BLASTP. The BLASTP results revealed 100 genes with a range of E values from 3.7E−32 to 6.2E−14 and similarities from 46.5% to 83.3% (Spreadsheet S1). Using the protein sequences of 100 rice genes and six *Arabidopsis* genes, a joint phylogenetic tree of all these genes in rice and *Arabidopsis* was constructed using the neighbour-joining method in the ClustalW2 software. On the sub-tree, 10 rice genes showed a close evolutionary relationship with the genes from the *Arabidopsis DREB1* family (Fig. S1). In contrast to other genes, all 10 genes were very similar to the *Arabidopsis DREB1s,* with the highest E values among the results of reciprocal blast searches in the *Arabidopsis* genome (Spreadsheet S1), suggesting that only these genes are the homologs of *Arabidopsis DREB1s.* Of these homologous genes, Os01g73770, Os02g45450, Os04g48350, Os06g03670, and Os06g06970 are located separately on rice chromosomes 1, 2, 4, and 6, respectively, but Os08g43200 and Os08g43210, or Os09g35010, Os09g35020, and Os09g35030 are distributed on same chromosomes in clusters.

The *DREB1* genes of rice and *Arabidopsis* were further analysed using ClustalW2 for multiple alignment and phylogenetic tree construction (neighbour-joining method). A multiple alignment of DREB1-related proteins of rice and *Arabidopsis* revealed that with the exception of Os06g06970, all rice *DREB1* genes retain the four conserved regions, i.e. the NLS, ERF/Ap2 domain, and the DSAW and LWSY motifs (Fig. 2B). In the phylogenetic tree, there were three sub-trees. The six *Arabidopsis* genes (the clustered genes *CBF1*–*CBF3* and the paralogous genes *DDF1*–*DDF2*) were clustered in a single sub-tree. The rice genes were grouped in two clusters, one of which contained two sets of tandemly distributed genes (Os08g43200 and Os08g43210, Os09g35010, Os09g35020, and Os09g35030) plus Os06g06970, whereas the other comprised four rice genes (Os01g73770, Os06g03670, Os02g45450, and Os04g48350). The phylogenetic analysis suggests that rice and *Arabidopsis DREB1* genes diverged after species differentiation between rice and *Arabidopsis*, and then increased by duplication (Fig. 2A).

Colinearity analysis of *DREB1s* from rice and *Arabidopsis* showed that each of the rice homologs possessed colinearity to *Arabidopsis DREB1s* to some extent. For easy description, colinearity analysis of all regions with *DREB1* homologs was performed both within and between the genomes of *Arabidopsis* and rice. In the *Arabidopsis* genome, fragments >100-kb long that contained either the *CBF1*–*CBF3* cluster on chromosome 4, or *CBF4* on chromosome 5, had four pairs of paralogs (i.e. AT4G25400/AT5G51780, AT4G25410/AT5G51790, AT4G25440/AT5G51980, or *CBF1*–*CBF3*/*CBF4*). The two unlinked regions on chromosome 1, either carrying *DDF1* or *DDF2*, had three pairs of paralogs (AT1G12560/AT1G62980, *DDF1*/*DDF2*, or AT1G12640/AT1G63050). All these results indicated that the clustered genes (*CBF1*–*CBF3*) and *CBF4*, equivalent to *DDF1* and *DDF2*, were paralogous to each other, and were likely derived from two rounds of segmental duplication. Furthermore, there was another pair of paralogs (AT5G51930–51950/AT1G12570) among these pairs of paralogous regions (*CBF1*-*4* and *DDF1*-*2*), suggesting that two pairs of paralogous or duplicated genes (*CBF1*–*CBF3* and *CBF4*, and *DDF1* and *DFF2*) came from an even earlier duplication in the *Arabidopsis* genome (Fig. S2; Spreadsheet S1).

Within the rice genome, there were six or five pairs of paralogs, respectively, in the 100–200 kb intervals containing each of the *DREB1* homologs on chromosomes 2 and 4 (i.e. Os02g45530/Os04g48400, Os02g45520/Os04g48390, Os02g45480/Os04g48375, Os02g45420/Os04g48330, Os02g45450/Os04g48350, or Os02g45380/Os04g48290), and on chromosomes 8 and 9 (i.e. Os08g43160/Os09g34950, Os08g43170/Os09g34960, cluster of Os08g43200–43210/cluster of Os09g35010–35030, Os08g43250/Os09g35600, and Os08g43270/Os09g35630) (Fig. S3). Among the above four regions and the fragment containing the *DREB1* homolog (Os06g03670) on chromosome 6, there were also four sets of paralogs: (Os02g45520/Os04g48390)/Os06g03860/Os09g34990, (Os02g45450/Os04g48350)/Os06g03670/(Os08g43200/Os08g43210)/(Os09g35010/Os09g35020/Os09g35030), Os06g03640/(Os08g43270/Os09g35630), and (Os02g45380/Os04g48290)/(Os08g43250/Os09g35600). Even though the members of the latter two sets were not found on chromosome 6 or chromosomes 2/4, the members on chromosome 6 of the former two paralogous sets (i.e. Os06g03670 and Os03860) were more similar to those on chromosomes 2/4 (i.e. Os02g45450/Os04g48350 and Os02g45520/Os04g48390) than on chromosomes 8/9 (i.e. Os08g43200–43210/Os09g35010–35030 and Os09g34990) (Fig. S3; Spreadsheet S1). All the results of colinearity analyses within the rice genome indicate that the five regions listed above are paralogous to each other to varying extents, and suggest that the pairs of paralogous segments on chromosomes 2/4 and 8/9 containing a higher density of paralog pairs were derived from recent segmental duplication, whereas the pairs of paralogous segments of chromosome 6 and chromosomes 2/4 originated from an earlier duplication.

**Figure 3:**
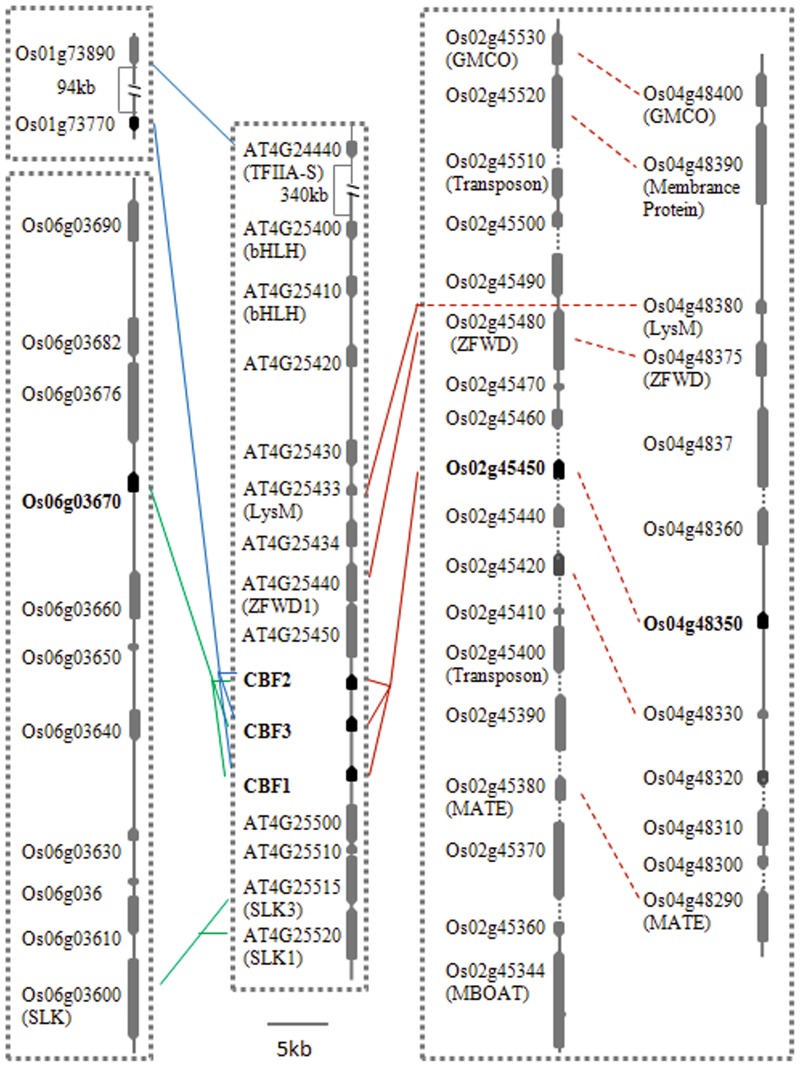
Colinearity among regions containing either *Arabidopsis CBF1–3* or their orthologs in rice. The dotted box indicates that the pairs of regions of chromosomes 2 and 4 contain lots of pairs of paralogs as showed by the dotted lines. The solid lines indicate that the genes are orthlogous to each other as they possess the highest E value or identities from reciprocal blast analyses.

After investigating the relationships between the paralogous regions within the rice or *Arabidopsis* genomes, we next analysed the colinearity of these regions between the rice and *Arabidopsis* genomes. The paralogous pair of fragments on rice chromosomes 2 and 4 had a total of three homologous genes on *Arabidopsis* chromosome 4 that contain *CBF1*–*CBF3* (i.e. AT4G25433/Os04g48380, AT4G25440/(Os02g45480/Os04g48375), *CBF1*–*CBF3*/(Os02g45450/Os04g48350). All homologs had the same transcriptional direction and arrangement order on the regions of *Arabidopsis* chromosome 4 and rice chromosomes 2 and 4. Located close to the homologous *CBF* gene (Os01g73770) on chromosome 1, Os01g73890 is one of two rice homologs of *Arabidopsis TFIIA-S* (AT4G24440) which also localise together to the *CBF1*–*CBF3* cluster. As a result, Os01g73770 is likely homologous to *DREB1* genes. The region bearing the *DREB1* homolog Os06g03670 on rice chromosome 6 had two pairs of homologs in the *Arabidopsis* region containing *CBF1*–*CBF3* with the same transcriptional direction and arrangement order (i.e. Os06g03670/*CBF1*–*CBF3*, Os06g03600/(AT4G25515–25520). Hence, in addition to Os01g73770, Os02g45450, and Os04g48350, Os06g03670 is also homologous to the *DREB1* genes (Fig. 3; Spreadsheet S1).

**Figure 4:**
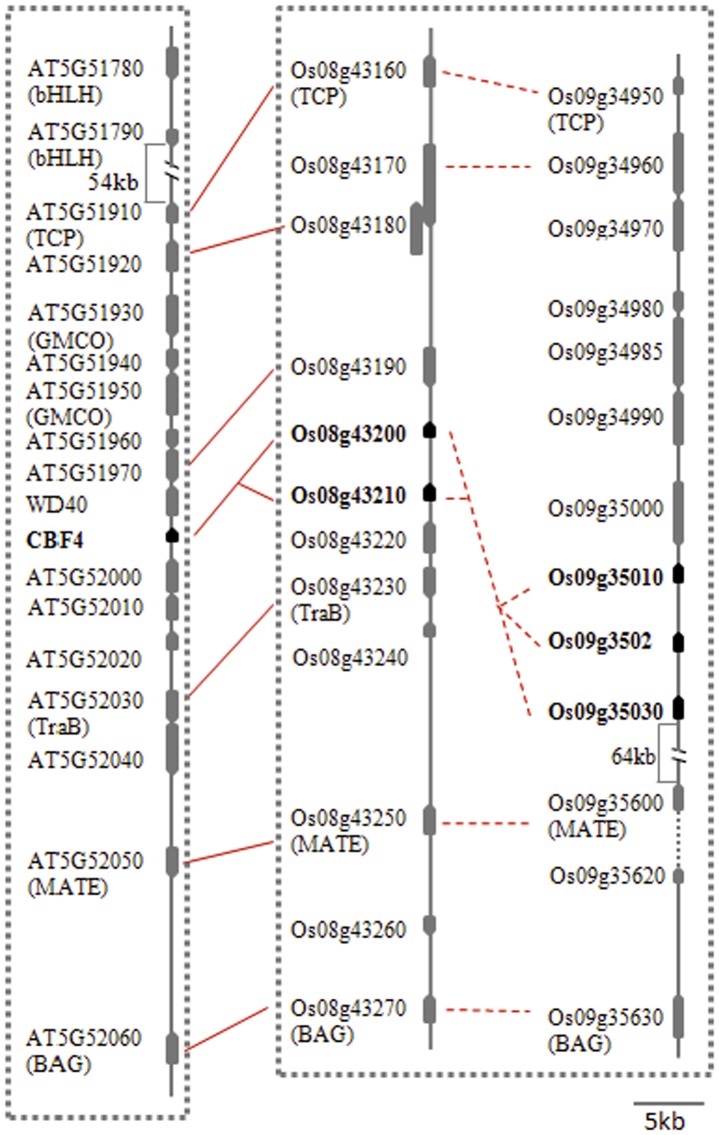
Colinearity among regions containing either *Arabidopsis CBF4* or its rice orthologs. The dotted box indicates that the pairs of regions of chromosomes 2 and 4 contain lots of pairs of paralogs as showed by the dotted lines. The solid lines indicate that the genes are orthlogous to each other, as they possess the highest E value or identities from reciprocal blast analysis.

The paralogous pair of clusters of Os08g43200–43210 and Os09g35010–35030 were mapped onto rice regions with perfect colinearity with the *Arabdopsis* region bearing *CBF4*, because the pair of paralogous regions on rice chromosomes 8 and 9 has a total of seven homologs to the following genes located in the region, including *CBF4* on *Arabidopsis* chromosome 5: AT5G51910/(Os08g43160/Os09g34950), AT5G51920/Os08g43180, AT5G517970/Os08g43190, *CBF4*/(Os08g43200–43210/Os09g35010–35030), AT5G52030/Os08g43230, AT5G52050/(Os08g43250/Os09g35600), and AT5G52060/(Os08g43270/Os09g35630) (Fig. 4; Spreadsheet S1). Thus, these genes are also likely to be homologs of *Arabidopsis DREB1* genes. This result was perfectly consistent with the results of a previous study by Vandepoele and colleagues [32].

The region containing the rice homolog Os06g06970, which is most similar to Os08g43200, did not show significant synteny to any *Arabidopsis* or rice regions containing *DREB1* genes, suggesting that it might be derived from single-gene duplication by the transposition of Os08g43200.

In summary, within the rice genome, rice regions containing Os02g45450, Os04g48350, Os06g03670, and the clustered genes Os08g43200–43210 and Os09g35010–35030 had some degree of colinearity with each other. Within the *Arabidopsis* genome, there was perfect colinearity between regions bearing the clustered genes *CBF1*–*CBF3* and *CBF4*, and regions containing *DDF1* and *DDF2*. Between the rice and *Arabidopsis* genomes, rice regions containing the homologs Os01g73770, Os02g45450, Os04g48350, and Os06g03670 had good colinearity to regions bearing *CBF1*–*CBF3*, whereas rice regions containing the paralogous pairs of clustered genes (i.e. Os08g43200–43210, and Os09g35010–35030) had good colinearity to the region bearing *CBF4*. Together with Os06g06970, the potentially transposed copy of Os08g43200, these rice genes were homologous to the *Arabidopsis DREB1* genes.

### Genome-wide Expression Analysis of Rice *DREB1* Genes

**Figure 5:**
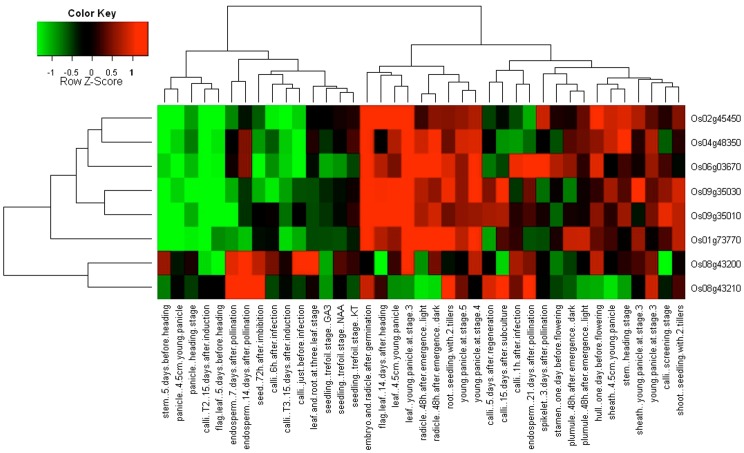
Expression pattern of rice *DREB1* genes in the ZS97 cultivar during the entire life cycle of the plant.

To evaluate the functional diversity of these rice *DREB1* genes, their expression patterns throughout the plant life cycle were examined using expression data from 39 tissues or treatments of the variety Zhenshan 97 from the CREP database. All rice homologs of *Arabidopsis DREB1* had cDNAs or expressed sequence tags (ESTs), except for Os09g35020, suggesting that Os09g35020 might be a pseudogene. Os06g06970 had a community cDNA and microarray probe, but the hybridization signal was too low to be detected (Table 1). Therefore, only the other eight genes were taken into account for genome-wide expression analysis (Fig. 5).

This analysis revealed that except for the cluster of Os08g432000 and Os08g43210, the other six genes were expressed in a similar pattern (Fig. 5). The levels of expression of the six genes were generally higher in young tissues (radicle, flag leaf, and young panicle) than in aged tissues (seed, endosperm, and stem). It was noteworthy that genes in the cluster Os09g35010/Os09g35030 or those in the pair of paralogs Os02g45450/Os04g48350 had the highest similarity. Two genes clustered on rice chromosome 8 Os08g43200/Os08g43210 also had a similar expression pattern, but this expression pattern was different from those of the other six genes. This result suggests that the six rice *DREB1s* (Os01g73770, Os02g45450, Os04g48350, Os06g03670, Os09g35010, and Os09g35030) have similar functions but differ functionally from the other two genes (Os08g48200 and Os08g43210).

### Expression of Rice *DREB1s* Under Abiotic Stresses

**Figure 6:**
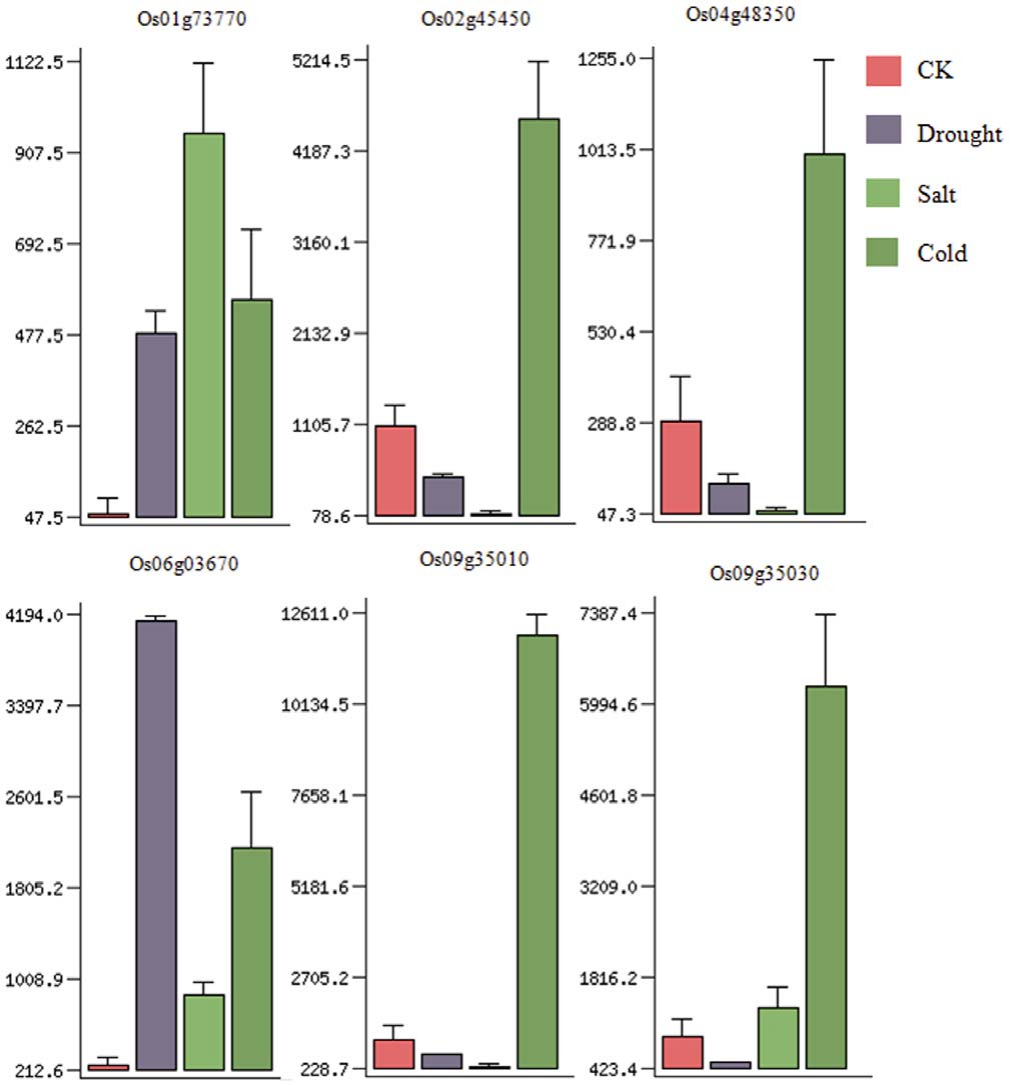
Expression of rice *DREB1s* under drought, salt, and cold stresses in rice seedlings from the SALK RiceGE database. X axis: different stresses; Y axis: total RNA expression level of seven-day-old seedlings.

To investigate the response of rice *DREB1s* in abiotic stresses, we compared the expression of all these genes in rice variety IR64 under three types of stress (drought, salt, and cold) using microarray data from RiceGE (Fig. 6). Two genes in the paralogous regions of chromosomes 2/4 (Os02g45450, Os04g45380), and those in the cluster of chromosome 9 (Os09g35010/Os09g35030) were massively and rapidly induced only by cold stress (4°C for 3 h). Os01g73770 and Os06g03670 were induced by all three abiotic stresses; moreover, the expression levels of Os01g73770 in response to salt stress and that of Os06g03670 in response to drought stress were much higher than those seen in response to other stresses. The other genes (Os06g06970, Os08g43200, Os08g43210, and Os09g35020) were not responsive to any of the stresses (data not shown).

### Real-time PCR Analysis of Rice *DREB1s* Under Cold Stress

**Figure 7:**
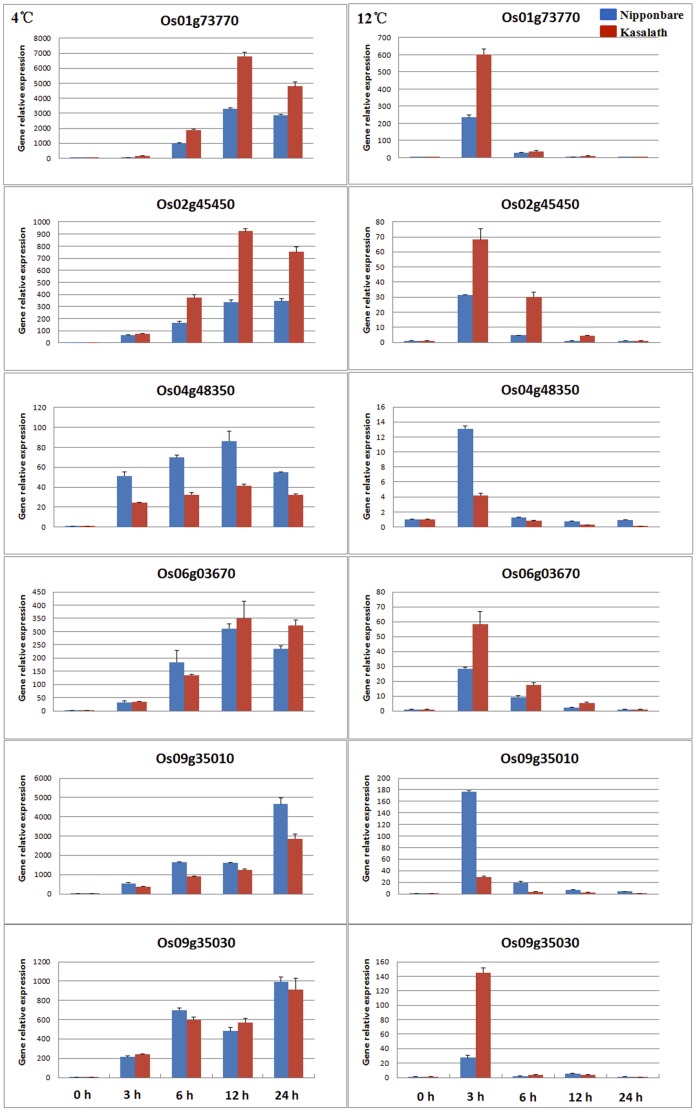
Expression of rice *DREB1*s under chilling condition (4°C) and chilling-acclimation (12°C) temperatures.

As most of the rice homologs of *Arabidopsis DREB1s* are known to be induced rapidly by cold stress, 7-day-old seedlings of two varieties, Nipponbare (japonica) and Kasalath (indica) were exposed to two different low temperatures (4°C or 12°C) to determine the expression pattern of the six rice *DREB1s* by quantitative reverse-transcription PCR (qRT-PCR; Fig. 7). The results indicated that all six genes were upregulated by the low temperature treatments, consistent with the microarray data. However, there were some differences between the two low temperature treatments. For all the genes, the expression level at 4°C was much higher and lasted longer than at 12°C. The expression level peaked at 6 h after treatment and lasted for more than 18 h at 4°C, with an increase of 80- to 7000-fold. In contrast, expression peaked at 3 h after treatment at 12°C, with an increase of 10- to 600-fold. Consistent with the microarray data, the results of qRT-PCR confirmed that the expression of four other rice *DREB1* homologs (i.e. Os06g06970, Os08g43200, and Os08g43210) did not change in response to cold stress (Fig. S4).

Through the japonica rice variety Nipponbare was more resistant to low temperatures than the indica rice variety Kasalath (Fig. 1), not all rice *DREB1s* showed higher expression levels under low temperature stress in Nipponbare than that in Kasalath. For example, upon treatment at both the low temperatures (4°C and 12°C), the expression levels of Os04g48350 and Os09g35010 were higher, whereas those of Os01g73770 and Os02g45450 were lower, in Nipponbare than in Kasalath. The expression levels of Os06g03670 and Os09g35030 were not significantly different between the two varieties at 4°C, whereas the level in Nipponbare was lower than that in Kasalath at 12°C (Fig. 7).

### GO Enrichment Analysis of co-expressed Genes of Each Rice *DREB1* Homolog

Genes with similar functions have similar expression profiles [33]. Co-expression has been widely used to identify genes involved in the same functional pathway [34], [35]. The fact that rice *DREB1*s are induced by cold treatment suggests that they may be involved in chilling acclimation or cold resistance in rice, just like their homologs in *Arabidopsis*. To confirm that the rice *DREB1* homologs are involved in the low temperature response, their co-expressed genes were used in GO enrichment analysis. The results showed that genes responsive to stress, especially those responsive to cold stress, were over-represented among the genes co-expressed with rice *DREB1* homologs (Table 2). The genes belonging to GO:006950 (responsive to stress) and/or GO:0009628 (responsive to abiotic stimulus) were obviously enriched among the genes co-expressed with Os01g73770, Os02g45450, Os04g48350, Os06g03670, Os09g35010, and Os09g35030. Moreover, most of the co-expressed genes in GO:0009628 (responsive to stress) were induced by cold stress (4°C for 3 h) according to expression profiles from the SALK RiceGE database. Further, GO:0009607 (responsive to biotic stimulus), GO:0009605 (responsive to external stimulus), and GO:0009719 (responsive to endogenous stimulus) were over-represented in the co-expressed genes of the above rice *DREB1* homologs. In the case of the rice *DREB1* homologs not induced by cold stress, such as Os08g43200 and Os08g43210, co-expressed genes belonging to GO:0006950 (responsive to stress) were obviously enriched, but did not respond to cold stress, according to expression data from the SALK RiceGE database. Therefore, Os08g43200 Os08g43210 might be involved in responses to stresses other than low temperature.

**Table 2:** GO enrichment analysis of co-expressed genes of rice *DREB1* homologs using agriGO software.

GO ID	Biological process	Total number of genes in the category	Number of co-expressed genes for each OsDREB1 (a),P-values of Fisher exact test for enrichment analysis (b),and number of cold-responsive genes (c).								
			Os01g73770	Os02g45450	Os04g48350	Os06g03670	Os09g35010	Os09g35030	Os08g43200	Os08g43210	
0006950	response to stress	1053	18^a^ (15^c^)	21 (15)	11 (7)	14 (12)	24 (19)	18 (17)	13 (1)	11 (2)	
			8.4E−11^b^	2.3E−12	4.2E−08	5.0E−08	1.0E−15	2.5E−10	3.7E−07	4.5E−06	
0009628	response to abiotic stimulus	67	10	6	10	8	8	12		7	
			4.9E−16	1.8E−08	6.2E−19	1.1E−12	4.0E−12	1.6E−19		4.0E−11	
0009607	response to biotic stimulus	20	6	15		6	11	5	7		
			3.2E−12	2.2E−35		1.8E−12	1.7E−24	8.6E−10	8.1E−15		
0009605	response to external stimulus	34	5	10		6	12	6			
			1.0E−08	1.2E−18		6.0E−11	1.3E−23	1.7E−10			
0009719	response to endogenous stimulus	49	13	15	7	14	11	11	9		
			7.3E−24	2.1E−27	1.2E−13	1.3E−26	2.7E−19	3.1E−19	9.0E−16		

To confirm the results of the co-expression analysis, GO enrichment analysis of the co-expressed genes of rice *DREB1* homologs was performed using the general and/or abiotic stress types from the ROAD database. The results were consistent with those of the analysis using CREP data, i.e. genes involved in the freezing response were over-represented among the genes co-expressed with Os01g73770, Os02g45450, Os04g48350, Os06g03670, Os09g35010, and Os09g35030, but not among those co-expressed with Os08g43200 and Os08g43210 (Table S1 and S2).

## Discussion

In our study, we have ascertained that rice possesses 10 homologs of *Arabidopsis DREB1* genes, which is consistent with previous reports that up to 10 DREB1-like genes exist in the rice genome [12, 13, and 14]. Skinner et al (2005) suggested that rice has at least 14 members of rice CBF family and ten of them were known as *OsDREB1A*-*OsDREBlJ* [13]. In our work, we showed that only these genes are the homologs of Arabidopsis DREB1s by reciprocal blast searches. However, how these genes evolved and how they respond to stress treatments is still not fully clear. The phylogenetic relationships and colinearity of DREB1-related regions within/between species indicate that rice and *Arabidopsis DREB1* genes diverged after the species differentiation of rice and *Arabidopsis*, and the family then expanded by proccesse such as segmental duplications, tandem duplications and transposition. Without considering single-gene duplication, the four paralogs of *Arabidopsis DREB1*s (*CBF1–CBF3*/*CBF4*/*DDF1*/*DDF2*) arose by three independent segmental duplications [36]. The six rice paralogs (Os01g73770/Os02g4540/Os04g48350/Os06g03670/Os08g43200–43210/Os09g35010–35030) might also be derived from several rounds of segmental duplication. Actually it has been confirmed that the four duplicated blocks, to which the pairs of *DREB1* homologous genes on chromosomes 2 and 4, and 8 and 9 were mapped, were derived from whole-genome duplication [37], [38].

Gene duplication plays an important role in biological evolution by supplying raw genetic material [39]. After duplication, the new gene descendants may maintain the same function as the parent genes, adopt part of the task of ancient genes (subfunctionalization), acquire novel functions (neofunctionalization), or change into nonfunctional pseudogenes (nonfunctionalization) [40]. In *Arabidopsis*, all of the tandem duplicated genes, *CBF1*–*CBF3*, function as key transcription factors in the cold-responsive pathway. The recently duplicated gene *CBF4* still functions in abiotic stress, but as a regulator of drought adaptation in *Arabidopsis* [41], whereas the much earlier duplicated genes, *DDF1* and *DDF2*, not only function in the response to salt stress but are also involved in plant development [42], [43].

Of the rice *DREB1* homologs, the recent segmentally duplicated genes, *OsDREB1E* and *OsDREB1G*, have the same expression pattern in the life cycle of the plant and both respond only to cold stress (Fig. 5 and 6). In the cluster of three *DREB1* genes on chromosome 9, *OsDREB1A* and *OsDREB1B* have the same expression pattern and both respond only to cold stress; therefore, these genes might retain the same function, whereas in the case of another tandem duplicated gene, *OsDREB1H*, no EST or cDNA can be found in the NCBI, KOME, or other databases, suggesting that it has become a nonfunctional pseudogene. In a previous study, Dubouzet et al reported that *OsDREB1A* was responsive not only to cold but also to salt stress [12], which is different from the results presented here and also the results of Ito et al (2006) (Fig. 6 [13]). Also, another paralog, *OsDREB1C*, was shown to be constitutively expressed, and not respond to individual stresses [12]. This discrepancy may be attributed to rice varieties with different genetic backgrounds or different methods employed to assay gene expression. In our study, *OsDREB1C* and its paralog *OsDREB1F* have expression patterns similar to those of other cold-responsive *DREB1*s, and are induced by other abiotic stresses in addition to cold stress, indicating that they retain a part of their functionality in the cold responsive pathway similar to *Arabidopsis CBF/DREB1s*. The paralogs containing two tandem duplicated genes on chromosome 8 (i.e. *OsDREB1I* and *OsDREB1J*,) may have gained a different function, because they have different expression patterns from those of cold-induced genes, and are not upregulated under any of the three stresses.

*CBF/DREB1*s of *Arabidopsis* and other species such as barley and wheat have been confirmed to play an important role in freezing resistance. For example, *CBF2* was the candidate of the major frost tolerant QTL (*FTQ4*) in a recombinant inbred line (RIL) population [44]. Frost resistance-2 (*FR-2*), a major QTL explaining up to 40% of the phenotypic variance, is localized to the same homologous genomic regions in different barley (*FR-H2*), or diploid (*FR-Am2*), and haploid (*FR-A2*) wheat mapping populations. Finally, both the major QTLs from the two different species are located in the *TaCBF* or *HvCBF* gene clusters, respectively, suggesting the importance of *CBFs* in freezing resistance [45, 46, 47, and 48]. In this study, the results of GO enrichment analysis indicated that six rice homologs of *DREB1*, all of which also responded to cold stress, had expression patterns similar to those of many other genes that are likely to be involved in the cold response. Moreover, the co-expressed genes included rice homologs of *Arabidopsis MYB15* [8] and *ZAT12* [9], both of which are key genes in the DREB1-dependent cold-responsive pathway (data not shown). Thus, the pathway may be conserved in rice to some extent, and rice *DREB1* may play an important role in cold-resistance.

Some QTLs related to cold or chilling tolerance have been mapped onto rice chromosomes using genetic populations, but few QTLs have been fine mapped to the regions containing *CBF/DREB1* genes [18, 19, 20, 21, 49, 50, 51, 52, 53, 54 and 55]. As we showed, rice possesses the phenomenon of chilling acclimation [Fig. 1], and the expression pattern of cold-responsive *OsDREB1*s under the acclimation temperature (12°C), rather than that under the chilling temperature (4°C), is similar to the expression pattern seen for *Arabidopsis CBF1–CBF*3 genes under cold acclimation temperature (4°C) [56], [57]. Thus for rice, acclimation under the mild temperature (i.e. 12°C), could have a positive effect on improving chilling tolerance, but the chilling temperature (i.e. 4°C.), which is effective for the acclimation of freezing-tolerant species, is harmful to chilling-sensitive plants. However, the above-mentioned rice QTLs were mapped by phenotypic identification without prior cold or chilling acclimation, and they may be loci involved only in cold resistance rather than chilling acclimation. Therefore, it is not surprising that they do not overlap with *DREB1* genes that may participate in chilling acclimation.

In this work, expression analysis of *DREB1* genes in the cold-tolerant japonica variety (Nipponbare) and the cold-sensitive indica variety (Kasalath) was performed, and the results showed no obvious relationship between the expression levels of *DREB1s* and the degree of cold resistance (Fig. 7). This is consistent with a report that *CBF* gene expression levels in *Arabidopsis* were not proportional with the degree of freezing tolerance [58]. This can be explained by the fact that besides the CBF/DREB1-dependent cold-responsive pathway, other pathways, such as the abscissic acid-dependent pathway, are involved in this process. The functional redundancy of *DREB1s* may attribute to the discrepancy. In addition, as rice usually grows in a warm environment, it is likely that it has just retained part of, rather than all, the functions of the cold response during its evolution, due to the accumulation of mutations. This is corroborated by the finding that *Arabidopsis* ecotypes growing in warmer latitudes generally exhibited lower expression levels of *CBF1*–*CBF3* [59].

In summary, as a prelude to elucidating the function of *DREB1*s in rice, we identified 10 homologs of *Arabidopsis CBF/DREB1*s, and found that some of these homologs are functionally conserved in the cold-responsive pathway. However, how these genes participate in cold signalling remains to be elucidated. Genetic analysis of single and multiple gene knockout mutants, and association mapping of *DREB1*s in natural rice populations, are underway in our lab. This research will facilitate our understanding of the molecular mechanisms of cold-responsive signalling in cold-sensitive plants.

## Supporting Information

Figure S1 **Phylogenetic tree of Arabidopsis CBF/DREB proteins and their homologs in rice, constructed using the neighbour-joining method of ClustalW2.**
(TIFF)

Figure S2 **Colinearity of** ***CBF/DREB1*** **regions within the** ***Arabidopsis*** **genome.** The solid lines indicate that the 2 genes have the highest E or identity values from BLASTP, while the dotted line indicates that the 2 genes possess some identity but do not have the highest E or identity values from BLASTP within the *Arabidopsis* genome. The presence of pairs of chromosomes regions within the same dotted box indicates that they are pairs of paralogous regions due to duplication.
(TIFF)

Figure S3 **Colinearity of rice** ***CBF/DREB1*** **regions within the rice genome.** The presence of 2 regions in the same dotted box indicates that they are a pair of paralogous regions due to segmental duplication, and the solid lines in the boxes indicate that the 2 genes have highest E or identity values from BLASTP. Outside the boxes, the solid and dotted unidirectional arrows indicate that the genes of chromosome 6 were used in BLASTP as queries, and those indicated by solid arrows have the highest E or/and identity values, compared to those indicated by the dotted arrows, with the exception of Os06g03640. The bidirectional arrow indicates that the 2 pairs of paralogs on chromosomes 2 and 4, and 8 and 9, have some degree of identity. Os06g03640 has the highest E and/or identity value from BLASTP with the pair of paralogs Os08g43270/Os09g35630.
(TIFF)

Figure S4 **Expression of rice** ***DREB1s*** **in the japonica rice variety Nipponbare under low temperature conditions (4°C).**
(TIF)

Table S1 **GO enrichment analysis of general ROAD co-expression data by the Chi square test.** a, total number of genes in the background from the ROAD database. b number of genes belonging to GO:0050826 (responsive to freezing). c total number of co-expressed genes of each rice DREB1 gene. d expected number of genes belonging to GO:0050826 of each rice DREB1’s co-expression genes. e number of co-expressed genes mapping to GO:0050826 (responsive to freezing). f total number of co-expressed genes mapping to GO:0050826 (responsive to freezing) and co-expressed genes induced by cold and mapping to GO:0006950 (responsive to stress). g, h values of chi square test. χ21, 0.05 = 3.84, χ21, 0.01 = 6.63.
(DOC)

Table S2 **GO enrichment analysis of the abiotic stress ROAD co-expression data using Chi square test.** a, total number of genes in the background from the ROAD database. b number of genes belonging to GO:0050826 (responsive to freezing). c total number of co-expressed genes of each rice DREB1 gene. d expected number of genes belonging to GO:0050826 of each rice DREB1’s co-expression genes. e number of co-expressed genes mapping to GO:0050826 (responsive to freezing). f total number of co-expressed genes mapping to GO:0050826 (responsive to freezing) and co-expressed genes induced by cold and mapping to GO:0006950 (responsive to stress). g, h values of chi square test. χ21, 0.05 = 3.84, χ21, 0.01 = 6.63.
(DOC)

Spreadsheet S1 **The blastp results of DREB1s and their neighbor genes within or between rice and Arabidopsis genomes.**
(XLSX)
