# Supplementary figures and images for: Colinearity and Similar Expression Pattern of Rice DREB1s Reveal Their Functional Conservation in the Cold-Responsive Pathway
Source: PLoS One. 2012 Oct 16;7(10):e47275. doi: 10.1371/journal.pone.0047275 (PMC3473061; doi:10.1371/journal.pone.0047275)

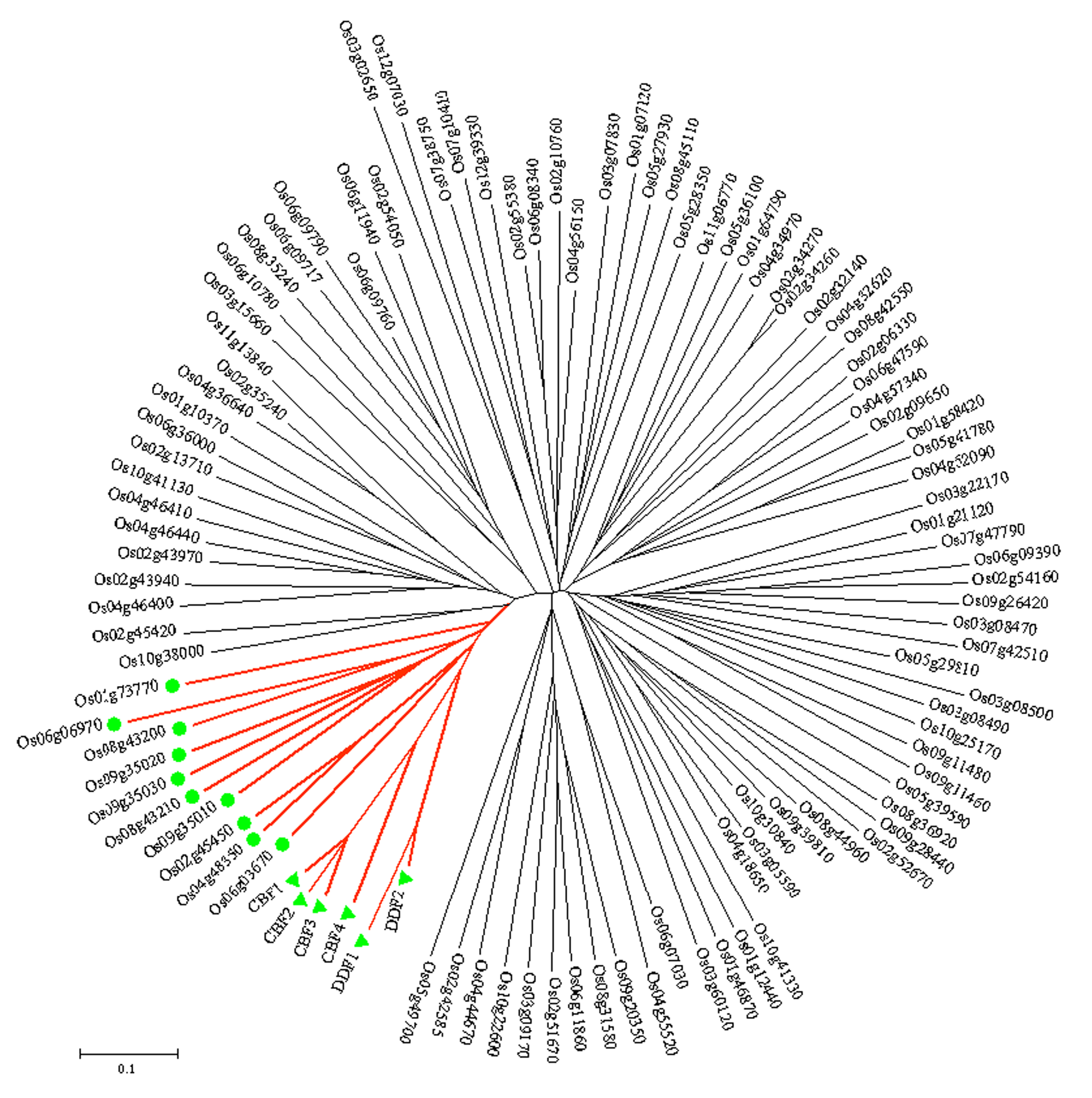

Supplement: Figure S1 — Phylogenetic tree of Arabidopsis CBF/DREB proteins and their homologs in rice, constructed using the neighbour-joining method of ClustalW2. (TIFF) [file pone.0047275.s001.tiff]

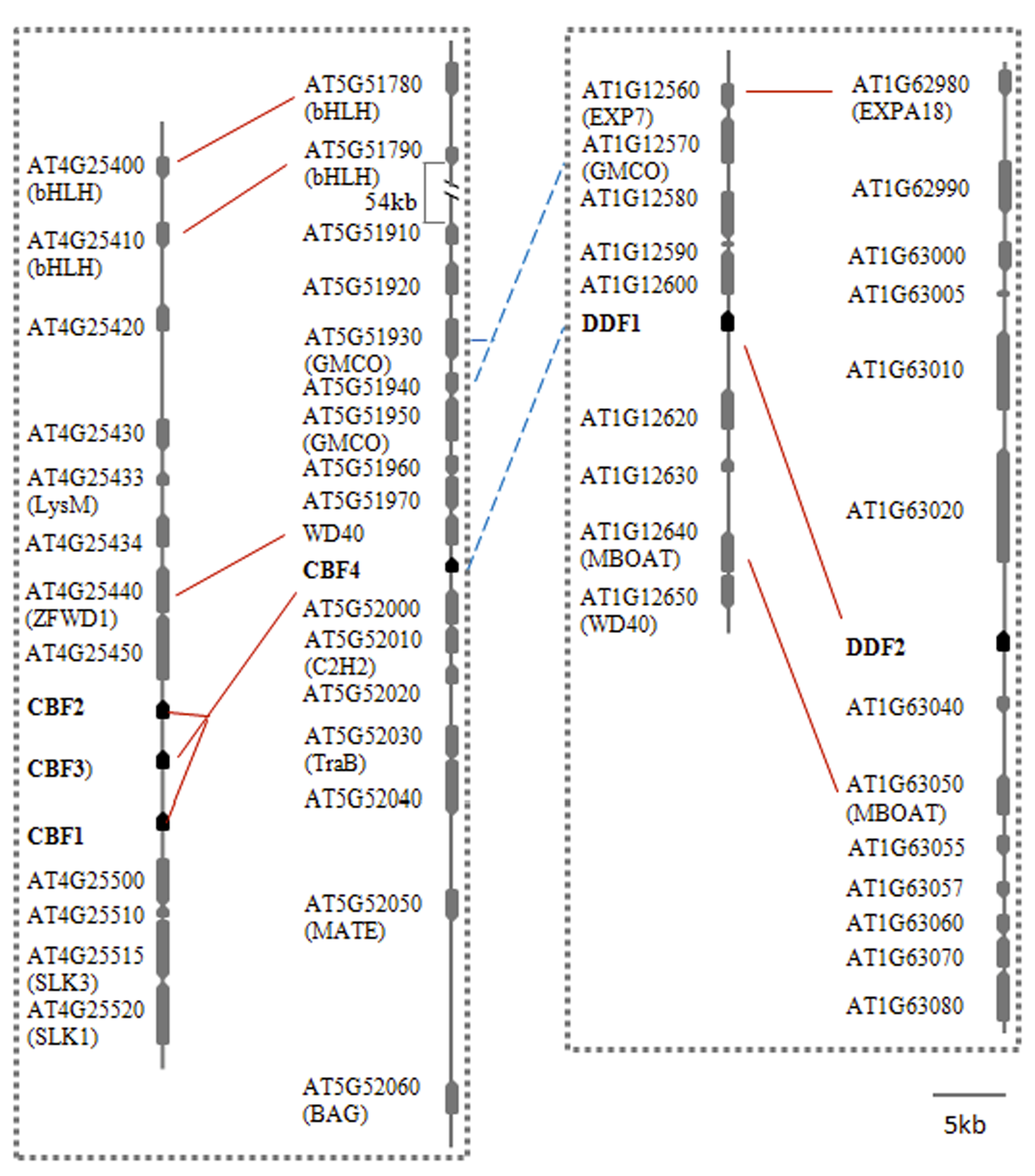

Supplement: Figure S2 — Colinearity of CBF/DREB1 regions within the Arabidopsis genome. The solid lines indicate that the 2 genes have the highest E or identity values from BLASTP, while the dotted line indicates that the 2 genes possess some identity but do not have the highest E or identity values from BLASTP within the Arabidopsis genome. The presence of pairs of chromosomes regions within the same dotted box indicates that they are pairs of paralogous regions due to duplication. (TIFF) [file pone.0047275.s002.tiff]

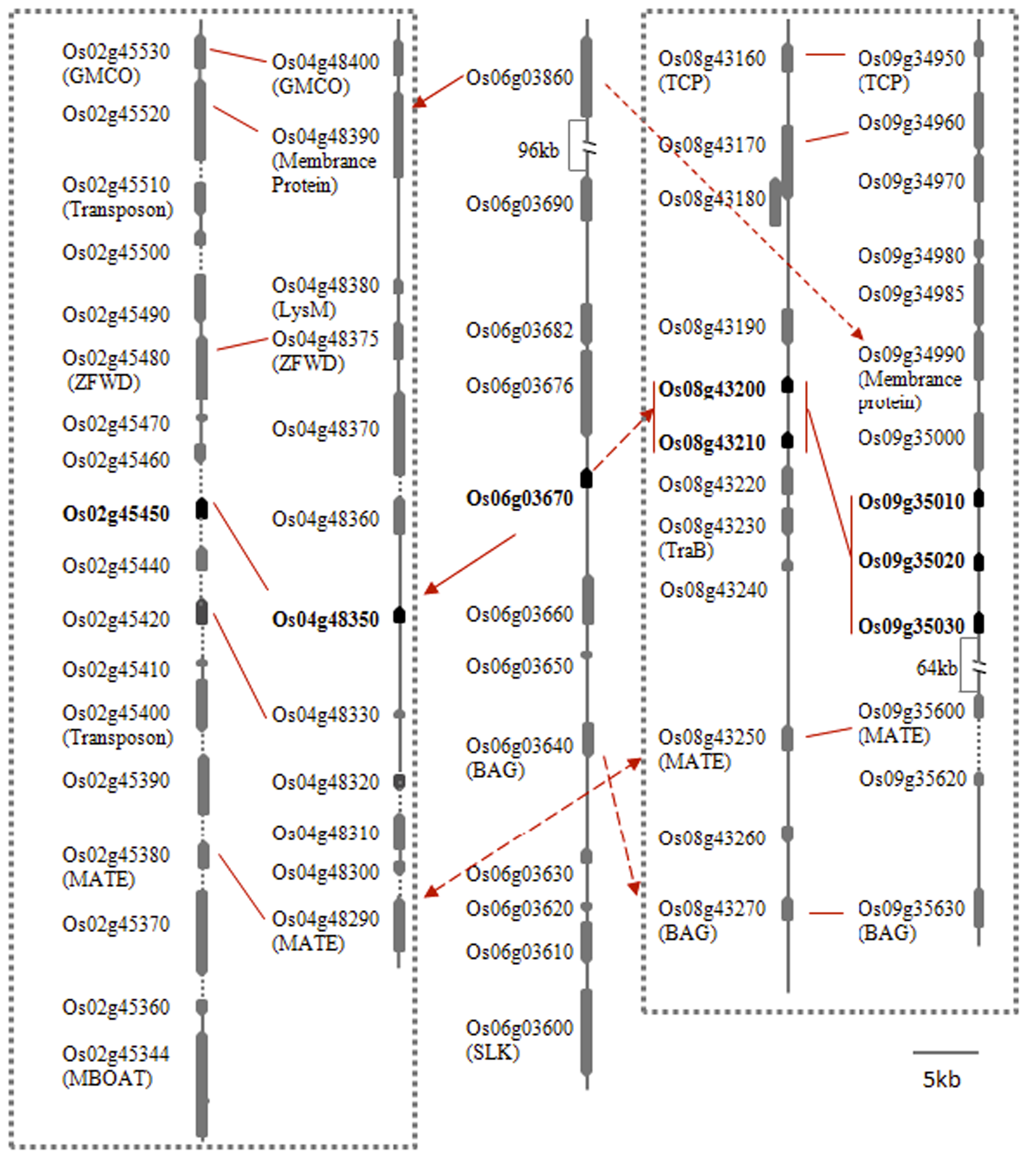

Supplement: Figure S3 — Colinearity of rice CBF/DREB1 regions within the rice genome. The presence of 2 regions in the same dotted box indicates that they are a pair of paralogous regions due to segmental duplication, and the solid lines in the boxes indicate that the 2 genes have highest E or identity values from BLASTP. Outside the boxes, the solid and dotted unidirectional arrows indicate that the genes of chromosome 6 were used in BLASTP as queries, and those indicated by solid arrows have the highest E or/and identity values, compared to those indicated by the dotted arrows, with the exception of Os06g03640. The bidirectional arrow indicates that the 2 pairs of paralogs on chromosomes 2 and 4, and 8 and 9, have some degree of identity. Os06g03640 has the highest E and/or identity value from BLASTP with the pair of paralogs Os08g43270/Os09g35630. (TIFF) [file pone.0047275.s003.tiff]

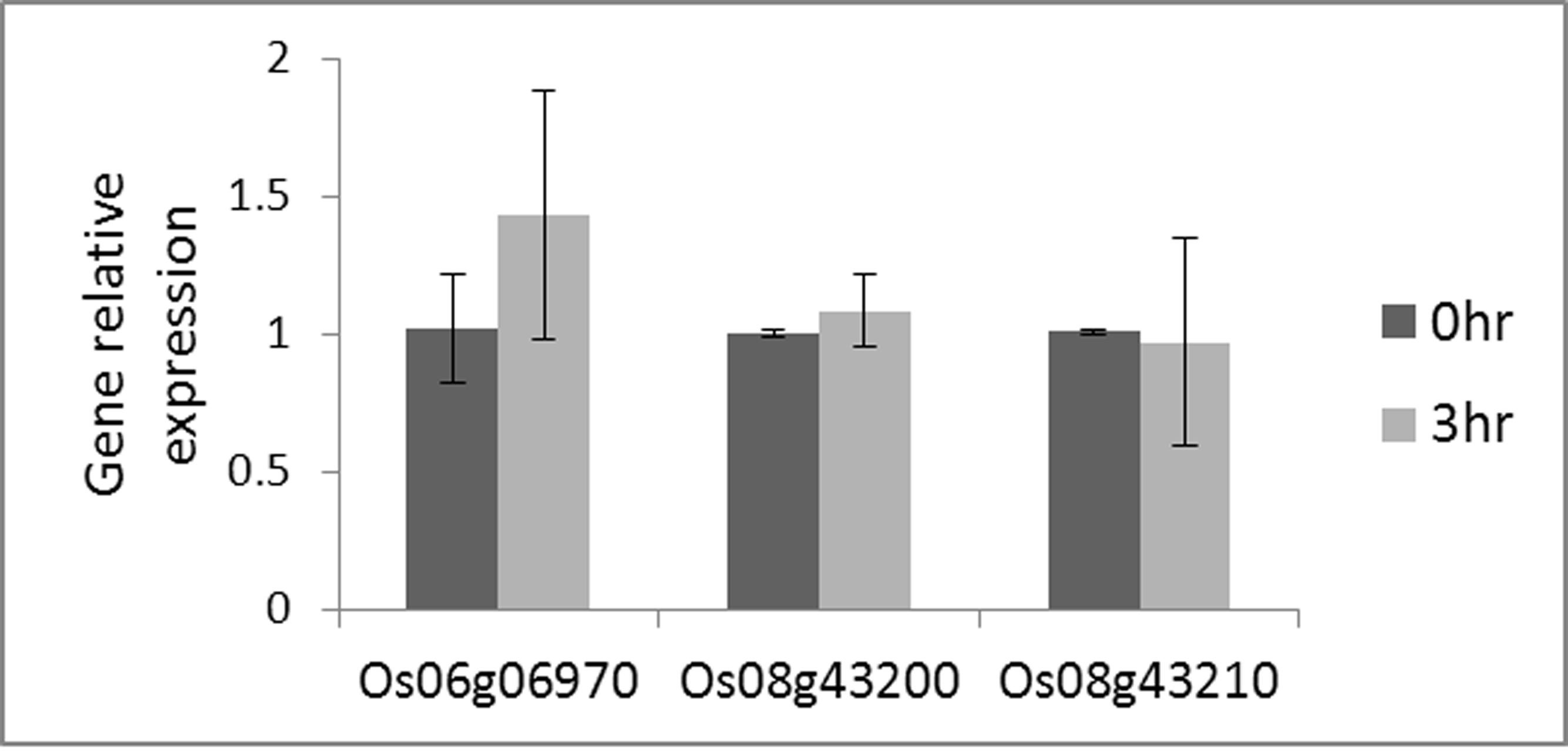

Supplement: Figure S4 — Expression of rice DREB1s in the japonica rice variety Nipponbare under low temperature conditions (4°C). (TIF) [file pone.0047275.s004.tif]
