# Supplementary material for: Colinearity and Similar Expression Pattern of Rice DREB1s Reveal Their Functional Conservation in the Cold-Responsive Pathway
Source: PLoS One. 2012 Oct 16;7(10):e47275. doi: 10.1371/journal.pone.0047275 (PMC3473061; doi:10.1371/journal.pone.0047275)
Supplement: Table S1 — GO enrichment analysis of general ROAD co-expression data by the Chi square test. a, total number of genes in the background from the ROAD database. b number of genes belonging to GO:0050826 (responsive to freezing). c total number of co-expressed genes of each rice DREB1 gene. d expected number of genes belonging to GO:0050826 of each rice DREB1’s co-expression genes. e number of co-expressed genes mapping to GO:0050826 (responsive to freezing). f total number of co-expressed genes mapping to GO:0050826 (responsive to freezing) and co-expressed genes induced by cold and mapping to GO:0006950 (responsive to stress). g, h values of chi square test. χ21, 0.05 = 3.84, χ21, 0.01 = 6.63. (DOC) [file pone.0047275.s005.doc]

**Table S**1. GO enrichment analysis of general ROAD co-expression data by the Chi square test

| OsDREB1s | Ref Total^a^ | Ref number^b^ | Query number^c^ | Query expect^d^ | Number of FZ^e^ | Number of CS^f^ | χ2_FZ^g^ | χ2_CS^h^ |
| --- | --- | --- | --- | --- | --- | --- | --- | --- |
| Os01g73770 | 39571 | 1647 | 143 | 6.0 | 12 | 21 | 5.48 | 37.32 |
| Os02g45450 | 39571 | 1647 | 162 | 6.7 | 9 | 19 | 0.51 | 21.55 |
| Os04g48350 | 39571 | 1647 | 74 | 3.1 | 7 | 13 | 4.07 | 30.34 |
| Os06g03670 | 39571 | 1647 | 171 | 7.1 | 12 | 26 | 2.88 | 49.77 |
| Os08g43210 | 39571 | 1647 | 36 | 1.5 | 0 | 0 | 2.69 | 2.69 |
| Os09g35010 | 39571 | 1647 | 192 | 8.0 | 14 | 26 | 4.03 | 40.22 |
| Os09g35030 | 39571 | 1647 | 201 | 8.4 | 16 | 27 | 6.43 | 41.21 |
