# Supplementary material for: Colinearity and Similar Expression Pattern of Rice DREB1s Reveal Their Functional Conservation in the Cold-Responsive Pathway
Source: PLoS One. 2012 Oct 16;7(10):e47275. doi: 10.1371/journal.pone.0047275 (PMC3473061; doi:10.1371/journal.pone.0047275)
Supplement: Table S2 — GO enrichment analysis of the abiotic stress ROAD co-expression data using Chi square test. a, total number of genes in the background from the ROAD database. b number of genes belonging to GO:0050826 (responsive to freezing). c total number of co-expressed genes of each rice DREB1 gene. d expected number of genes belonging to GO:0050826 of each rice DREB1’s co-expression genes. e number of co-expressed genes mapping to GO:0050826 (responsive to freezing). f total number of co-expressed genes mapping to GO:0050826 (responsive to freezing) and co-expressed genes induced by cold and mapping to GO:0006950 (responsive to stress). g, h values of chi square test. χ21, 0.05 = 3.84, χ21, 0.01 = 6.63. (DOC) [file pone.0047275.s006.doc]

**Table S**2. GO enrichment analysis of the abiotic stress ROAD co-expression data using Chi square test

| OsDREB1s | Ref Total^a^ | Ref number^b^ | Query number^c^ | Query expect^d^ | Number of Fz^e^ | Number of CS^f^ | χ2_FZ^g^ | χ2_CS^h^ |
| --- | --- | --- | --- | --- | --- | --- | --- | --- |
| Os01g73770 | 39571 | 1647 | 39 | 1.6 | 6 | 6 | 9.90 | 9.90 |
| Os02g45450 | 39571 | 1647 | 79 | 3.3 | 7 | 12 | 3.37 | 21.63 |
| Os04g48350 | 39571 | 1647 | 19 | 0.8 | 2 | 5 | 0.80 | 18.62 |
| Os06g03670 | 39571 | 1647 | 355 | 14.8 | 14 | 25 | 0.11 | 6.74 |
| Os08g43210 | 39571 | 1647 | 200 | 8.3 | 9 | 11 | 0.01 | 0.62 |
| Os09g35010 | 39571 | 1647 | 176 | 7.3 | 13 | 20 | 3.88 | 21.26 |
| Os09g35030 | 39571 | 1647 | 534 | 22.2 | 28 | 36 | 1.33 | 8.33 |
